# Multidrug-Resistant Bacteria from Raw Meat of Buffalo and Chicken, Nepal

**DOI:** 10.1155/2019/7960268

**Published:** 2019-05-02

**Authors:** Bhuvan Saud, Govinda Paudel, Sharmila Khichaju, Dipendra Bajracharya, Gunaraj Dhungana, Mamata Sherpa Awasthi, Vikram Shrestha

**Affiliations:** ^1^Department of Medical Laboratory Technology, Janamaitri Foundation Institute of Health Sciences (JFIHS), GPO Box 8322, Hattiban, Lalitpur, Nepal; ^2^Department of Nursing, Janamaitri Foundation Institute of Health Sciences (JFIHS), GPO Box 8322, Hattiban, Lalitpur, Nepal

## Abstract

Antimicrobial resistance is a major global issue for human and animals. Increased use of antimicrobials in livestock and poultry has become one of the causes of antimicrobial resistance development in microorganisms. The aim of the study was to characterize antimicrobial resistant bacteria from raw buffalo and chicken meat in standard* in vitro* condition. A total of 140 raw meat samples were collected from different retail shops of Bhaktapur Metropolitan City, Nepal. Among them, 70 were raw buffalo meat and 70 were raw chicken meat samples. Bacterial growth, identification, and antimicrobial susceptibility test were performed according to Clinical & Laboratory Standards Institute (CLSI) guidelines. Out of 140 samples, bacterial growth was seen in 67 raw buffalo meat and 59 raw chicken meat samples, i.e., bacterial growth was observed in 90.0% of the samples. A total of 161 bacterial isolates were detected.* Escherichia coli* (35.4%) and* Klebsiella *spp. (30.4%) were found to be the most prevalent bacteria followed by* Citrobacter *spp. (11.8%),* Staphylococcus aureus* (9.3%),* Salmonella *spp. (7.4%), and* Proteus *spp. (5.5%). Chicken meat isolates showed higher antimicrobial resistance rates in comparison to buffalo meat isolates, particularly against antimicrobials like Amoxicillin, Tetracycline, Cotrimoxazole and Nalidixic acid,* p* value<0.05 when compared between buffalo and chicken meat. Overall, 32.7% Multidrug-Resistant (MDR) isolates were found, in which 50.0% MDR isolates were found from chicken raw meat and 21.9% were found from buffalo raw meat. MDR isolates of* Escherichia coli*,* Proteus *spp. and* Staphylococcus aureus* constituted 52.5%, 77.7% and 40.0%, respectively, of both buffalo and chicken raw meat. This study indicates antimicrobials resistant bacteria existing at an alarming rate, higher in chicken meat than in buffalo meat.

## 1. Introduction

Burden of antimicrobial resistant bacteria affects the economy and health of people in both developed and developing countries. Globally, bacterial antimicrobial resistance is witnessing a rapid rise in both human and veterinary world [1, 2]. Antimicrobials are becoming increasingly ineffective and are posing one of the biggest threats to both humans and animals [3]. It was reported that common antimicrobial resistant bacteria like* Escherichia coli*,* Klebsiella pneumoniae*,* Staphylococcus aureus*,* Streptococcus pneumoniae*, and* Salmonella *spp., that are the common human normal microbiota as well as important human pathogens, are the major flora among foodborne pathogens [4, 5]. Poultry and livestock are reservoir of such drug-resistant microorganisms and are promoting the growth and dissemination of pathogens as well as drug resistance [6]. Food of animal origin that carry a variety of pathogenic and nonpathogenic bacteria have become a platform that provides an environment for interactions that might lead to the evolution of new drug-resistant and Multidrug-Resistant (MDR) bacteria through the means of horizontal exchange of drug-resistant genes [7, 8]. The extensive use of antimicrobials in the poultry industry for disease prevention and as growth promoter further triggers the mechanisms that lead to the emergence of drug-resistant strains of bacteria.

Globally, poultry farming sector continues to become industrialized in order to meet the consumers' demand. Nepal ranks 112th in the sector of meat production from poultry. It contributes to around 3.5% of Gross Domestic Product (GDP). The production of poultry meat in half-decades from 2005/6 to 2010/11 saw an increment of more than twofold, from 15835 tons to 36303 tons. Likewise, the production of buffalo meat slightly increased from 122040 tons to 167868 tons during the same time period [9]. In order to meet the consumers' increasing demand of meat from poultry and livestock, the farmers resort to the excessive use of antimicrobials as one of the most accessible and prompt ways to prevent animal diseases and thereby increase the production [10]. Excessive and irrational use of antimicrobials in the name of increasing production simultaneously has an impact on the microbiological world that leads to the circumstances causing the evolution and development of antimicrobial resistance on bacterial flora. The newly emerged drug-resistant bacteria, hence, are better equipped to act as pathogens for those working in farm, animal health sector, and meat store and consumers through direct and indirect contact via food product, contact with infected animals, through manure etc.

Majority of Nepalese population consume meat products processed in small shops that store the product at ambient temperature and improper handling, both of which increase microbial growth and contamination. A study showed that environmental conditions of meat shops of Kathmandu were unsatisfactory and more than 80.0% meat were contaminated with coliform bacteria [11]. Similarly, 85.6% of slaughtering places in Pokhara, a tourism city in Nepal, were not hygienic [12]. In addition to these two factors, the practice of sale of veterinary drugs without the consultation and prescription of veterinary healthcare workers boost the chances of antimicrobial resistance evolution in pathogens [13]. For the feed supplement, 50.0% antimicrobials were prescribed inappropriately and about 71.0% veterinary drugs were sold with self-prescription without suggestion of qualified registered veterinarian [13]. In Nepal, antimicrobial resistance patterns are poorly documented and only limited studies are available on bacterial prevalence and their antimicrobial resistance pattern [13, 14]. Nepal currently lacks a government level veterinary antimicrobial resistance surveillance network and veterinary drug use regulations and guidelines. However, Global Antibiotic Resistance Partnership (GARP), Nepal working group is working with the World Health Organization (WHO) for the development of national action in order to work collaboratively with the government and nongovernment partners [13]. Therefore, this present study is designed to investigate the prevalence of antimicrobial drug-resistant bacteria from raw meat sample from Bhaktapur, an ancient city in Nepal.

## 2. Materials and Methods

### 2.1. Study Design, Study Area and Sampling

A cross-sectional prospective study was carried out in Bhaktapur Metropolitan City from April to September 2017. 140 raw meat samples were collected from the retail shops, where 70 were raw buffalo meat and 70 were raw chicken meat. Simple random sampling technique was employed where both fresh and frozen raw meat samples were collected aseptically from randomly selected retail meat shops in Bhaktapur. The meat products were placed in sterile, leakproof container in cold chain box. The samples were transported to the Department of Medical Laboratory Technology, JF Institute of Health Sciences (JFIHS), Hattiban, for further investigation.

### 2.2. Bacterial Isolation and Characterization

25 grams of meat sample were homogenized for 2 minutes with 250 ml of 1% buffered peptone water. 0.1 ml of sample was transferred onto each of Blood Agar (Hi-Media, India), MacConkey Agar (Hi-Media, India), and Mannitol Salt Agar (Hi-Media, India). Media and the plates were incubated for 24 hours at 37°C. The colonies were further identified by their morphological characteristics, staining characteristics, and biochemical properties as described by protocols of Clinical and Laboratory Standards Institute (CLSI) [15].

### 2.3. Antimicrobial Susceptibility Test

The antimicrobial susceptibility of bacterial isolates was performed by using Kirby Bauer Disk diffusion method on Mueller Hinton Agar (Hi-Media, India) according to the CLSI guidelines [15]. The antibiotic susceptibility pattern was examined by using commercial antibiotic discs including Amoxicillin (10*μ*g), Ceftazidime (30*μ*g), Gentamicin (10*μ*g), Tetracycline (30*μ*g), Nitrofurantoin (300*μ*g), Cotrimoxazole (1.25*μ*g), Nalidixic acid (30*μ*g), Cefoxitin (30*μ*g), Azithromycin (15*μ*g), and Ciprofloxacin (5*μ*g) (Hi-Media, India). The* Escherichia coli* isolate ATCC 25922 and* Staphylococcus aureus* isolate ATCC 25923 were used as reference organisms for quality control to antimicrobial susceptibility testing.

### 2.4. Statistical Analysis

Data were analyzed by using Statistical Package for the Social Sciences (SPSS, Chicago, IL, USA) version 20 and interpreted according to frequency distribution and percentage. Independent* t*-test was used for analysis of data and* p* value of <0.05 was considered statistically significant.

## 3. Results

The present study included a total of 140 raw meat samples of buffalo and chicken. Among them, 70 samples were raw chicken meat and 70 samples were raw buffalo meat. 67 samples of buffalo meat showed bacterial growth, whereas 3 samples did not show any growth. Likewise, 59 chicken meat samples showed bacterial growth and 12 samples did not show any growth in standard* in vitro* condition.* Escherichia coli* was the most commonly found bacterial isolate from both buffalo and chicken meat, i.e., 31.6% and 33.0%, respectively. Subsequently, another commonly found bacterium was* Klebsiella *spp., the prevalence of which was 32.9% in buffalo meat and 24.0% in chicken meat followed by* Citrobacter *spp. 10.5% in buffalo meat and 11.0% in chicken meat. Similarly,* Staphylococcus aureus* was 7.9% and 9.0%, respectively, in buffalo meat and chicken meat.* Salmonella *spp. and* Proteus *spp. were isolated in lesser number compared to other bacterial isolates as shown in Table 1.

Higher phenotypic expression of antimicrobial resistance and subsequently higher number of multidrug-resistant bacteria were found in chicken raw meat in comparison to buffalo raw meat samples.* Escherichia coli* was found to be the most predominant organism in meat samples of both buffalo and chicken. Out of 33* Escherichia coli* isolates from chicken raw meat, resistance to Amoxicillin, Tetracycline, and Nalidixic acid was 69.6%, 60.6% and 54.4%, respectively. Buffalo meat isolates showed resistance rate of 41.6% to Tetracycline followed by 16.6% to Amoxicillin, 16.6% to Cotrimoxazole, and 12.5% to Gentamycin. Out of 24* Klebsiella *spp. isolated from chicken raw meat, 79.1% were resistant to Amoxicillin, 41.6% to tetracycline, 33.3% to Nalidixic acid, and 29.1% to Cotrimoxazole. In buffalo meat out of 25* Klebsiella *spp. isolated, 64.0% were resistant to Amoxicillin, 16.0% to Nitrofurantoin, and 12.0% to Tetracycline. Among 6* Salmonella *spp. isolated from chicken, only one isolate was resistant to Amoxicillin. Out of 11* Citrobacter* spp. isolated from chicken, 54.5% were Amoxicillin resistant and 18.8% were Tetracycline resistant. But in buffalo meat, only 25.0%* Citrobacter *spp. were amoxicillin resistant out of 8 isolates shown in Table 2. Similarly, among* Staphylococcus aureus* isolates from chicken, 44.4% isolates were resistant to Tetracycline, 33.3% were resistant to Amoxicillin, and 22.2% were resistant to Azithromycin. From buffalo meat, out of 6 isolates of* Staphylococcus aureus*, 50.0% were Amoxicillin resistant, 50.0% were Cotrimoxazole resistant and 50.5% were resistant to Tetracycline. Resistance to the rest of the antimicrobials was not seen.

Comparison of drug resistance among isolates from raw buffalo and chicken meat showed a statistically significant difference in resistance to Amoxicillin, Tetracycline, Cotrimoxazole and Nalidixic acid,* p* value < 0.05. Chicken meat showed larger number of MDR isolates. Resistance to at least 3 or 4 and > 4 drugs was significantly higher in isolates from chicken raw meat in comparison to isolates from buffalo meat,* p* value <0.05, as shown in Table 3.

A total of 161 bacterial isolates were detected from the 140 samples. Out of 161 bacterial isolates, 32.7% were MDR. Among them, the MDR bacteria from chicken were higher than from buffalo meat, 50.0% and 21.9%, respectively. In raw chicken meat, out of 33 isolates, 69.6% were MDR bacterial isolates of* Escherichia coli*. Similarly, out of 24 isolates of* Klebsiella *spp., 45.8% isolates were MDR resistant and of* Proteus *spp. all the isolates were MDR isolates. In gram positive bacteria, MDR isolates were 50.0% and 33.0%, respectively, from buffalo and chicken*. Staphylococcus aureus *isolates from buffalo were resistant to Amoxicillin, Tetracycline, and Cotrimoxazole and isolates from chicken were resistant to Amoxycillin, Tetracycline, and Azithromycin. No Methicillin-Resistant* Staphylococcus aureus* (MRSA) was found in this study. In Buffalo meat, out of 73 bacterial isolates, 21.9% were MDR isolates; out of 24* Escherichia coli,* 29.5% were MDR. Out of 25* Klebsiella *spp., 16.0% were MDR isolates and out of 6* Staphylococcus aureus, *50.0% were MDR isolates. In both buffalo and chicken meat*, Salmonella *spp. was found to be sensitive to all antimicrobials tested. Of 19 total* Citrobacter *spp. isolated, 10.5% were MDR, all of which were chicken isolates, i.e., no MDR isolates from buffalo meat were detected as shown in Table 4.

## 4. Discussion

In the present study, out of 140 samples, 90.0% samples had bacterial growth in different culture medium. The growth of bacteria was higher in buffalo meat samples (95.7%) as compared to chicken meat (84.0%). A similar finding was reported from Kathmandu, where they found more than 80.0% of sample had coliform bacteria [11]. A similar finding was reported from Pakistan, where they found 84.0% samples contaminated with different bacterial species, among which 66.0% bacterial isolates were potential pathogens [16]. The high level of bacterial growth was due to the high contamination of the meat after slaughter and the long storage at ambient temperature.

*Escherichia coli* was the most commonly detected bacteria in our study with a high prevalence of 31.6% in buffalo meat and 33.0% in chicken meat. A study from Nepal showed that prevalence of* Escherichia coli* was found to be 66.6% and 40.0% in chicken and buffalo meat, respectively [17]. Antimicrobial resistant* Escherichia coli* from chicken meat were also reported from different parts of the world including 40.6% in Japan [18], 52.0% in Iceland [19], and 83.8% in Vietnam [20].* Escherichia coli *is an established normal microflora of gastrointestinal tract of chicken and livestock. In our study, out of 57 isolates of* Escherichia coli* from both chicken and buffalo meat, 30 were MDR. In comparison of buffalo and chicken meat, a higher number of MDR strains were found from chicken meat sample. From chicken meat, 69.7% MDR strains were isolated. In poultry, it was reported that intestinal microflora changed into MDR, 77.4% from Saudi Arabia [21], 81.3% from households and small-scale farms in Vietnam [22]. A study conducted in chicken breast sample in the United States showed 83.5% prevalence of* Escherichia coli*, of which 38.9% isolates were MDR [23]. High number of* Escherichia coli* in retail meats indicates fecal contamination at slaughter or during processing. In our study,* Klebsiella *spp. was the second highest prevalent organism, 32.9% in buffalo and 24.0% in chicken meat, mainly resistant to Amoxicillin and Tetracycline. From the total isolates of* Klebsiella* spp., 33.6% isolates were MDR. Out of the total isolates from chicken meat, 45.8% were MDR and out of the total isolates from buffalo, 16.0% were MDR. Similar finding was reported from South Africa in which in chicken around 40.0% isolates were MDR [24] and from Egypt, 35.0% of broilers' internal organs were positive for* Klebsiella pneumoniae* [25].

Interestingly, in this study,* Salmonella *spp. isolates from buffalo meat were sensitive to all antimicrobials, whereas 16.6% of isolates from chicken meat were resistant to Amoxicillin with no MDR isolates. But findings contrasting to ours were reported from Nepal, Turkey, and China where MDR isolates were 100%, 81.1% and 100%, respectively [26–28].* Salmonella *spp. is considered as a major foodborne pathogen due to cross contamination and undercooked meat eating habit. Our study showed that out of 15* Staphylococcus aureus* isolated, 6 were multidrug resistant, mainly resistant to Amoxicillin and Tetracycline. No MRSA strains were found. In chicken meat,* Staphylococcus aureus* prevalence was higher than that in buffalo meat with a prevalence of 9.0% and 7.9%, respectively. Dissimilar results, in previous investigation on chicken meat, were found to be 75.0% in Bangladesh [29] and 18.2% in the United States [30]. MRSA in chicken meat was found to be 20.0% in Bangladesh [29] and 25.0% from fresh chicken in Germany [31]. Results similar to ours were reported from Austria [32].* Citrobacter *spp. were isolated in 10.5% of buffalo meat and 11.0% chicken meat. Among them, multiple antimicrobials resistance was found in those isolated from chicken. Amoxicillin-resistant isolates in buffalo and chicken meat were found to be 25.0% and 54.5%, respectively. Out of total 19 isolates, 10.5% were MDR and all were chicken meat isolates. A study from Indonesia showed 10.8% prevalence of* Citrobacter *spp. with 63.6% MDR isolates [33]. A study from Nepal showed* Citrobacter *spp. isolates to have a prevalence of 44.7% with 7.8% MDR isolates [34].* Proteus *spp. was less prevalent compared to other bacterial isolates having a prevalence of 5.3% and 5.0% prevalence in buffalo and chicken samples, respectively. Buffalo meat had 50.0% MDR isolates, whereas 100% of the isolates from chicken meat sample were MDR. Similar finding was reported from Iran in chicken meat sample, where 96.0% isolates were resistant to two or more than two antimicrobials [35]. Isolates from chicken meat were significantly resistant to antimicrobials like Amoxicillin, Tetracycline, Cotrimoxazole, and Nalidixic acid in comparison to those from buffalo meat,* p* value <0.05. Isolates from buffalo meat showed higher resistance in none of the antimicrobials compared to chicken meat. Among the MDR cases, chicken isolates had significantly higher number of resistance to 3 to 4 drugs as well as more than 4 drugs in comparison to those from buffalo meat,* p* value <0.05. A research from the United States also found significant difference on Tetracycline resistance in poultry and beef meat [36]. The reason behind this might be due to excessive use of antimicrobials in chicken feed and the environment where chicken are raised.

The bacterial isolates we detected are all common human pathogens that can not only play role in drug resistance dissemination, but also potentially cause serious human infections of various kinds like urinary tract infection, septicemia, pyogenic infections, and other illnesses. These pathogens are the source of infection to all the population in community as each one of us is directly or indirectly related to meat product production, processing or consumption. All the bacteria we have isolated are merely a few of the indicators of meat product contamination. The threat of biological hazard brought about by the meat products can be grossly argued under the major issues like irrational antimicrobial use, inappropriate processing setting, traditional eating habit of uncooked or undercooked meat, lack of public awareness of basic hygiene habits in general, and lack of proper access to health facility. In Nepal, 92.6% of population are nonvegetarian, which means a huge proportion of population are at risk of meatborne hazards [37].

## 5. Conclusion

In conclusion, the result of this study provides preliminary data on antimicrobial resistant bacteria from raw buffalo and chicken meat of Bhaktapur. From the study, it is evident that meat products are biologically unsafe for consumption. Extensive researches on this issue that also involve the molecular dynamics should be conducted longitudinally to have a better understanding of the exact scenario throughout the nation and thereby help curb the possible threats. Training and awareness program should be conducted in order to minimize the irrational use of antimicrobials and hence reduce drug resistance evolution via poultry and livestock.

## Figures and Tables

**Table 1:** Bacterial prevalence from raw meat of buffalo and chicken.

Bacteria	Buffalo meat		Chicken meat	
	Count(n)	Prevalence (%)	Count(n)	Prevalence (%)
*Escherichia coli*	24	31.6%	33	33.0%
*Klebsiella *spp.	25	32.9%	24	24.0%
*Salmonella *spp.	6	7.9%	6	6.0%
*Citrobacter *spp.	8	10.5%	11	11.0%
*Proteus *spp.	4	5.3%	5	5.0%
*Staphylococcus aureus*	6	7.9%	9	9.0%
No growth	3	3.9%	12	12.0%

**Table 2:** Antimicrobial resistance properties of Enterobacteriaceae isolates from samples.

Antimicrobials	*Escherichia coli*		*Klebsiella *spp.		*Salmonella *spp.		*Citrobacter *spp.		*Proteus *spp.	
	Buffalo meat	Chicken meat	Buffalo meat	Chicken meat	Buffalo meat	Chicken meat	Buffalo meat	Chicken meat	Buffalo meat	Chicken meat
	(n=24)	(n=33)	(n=25)	(n= 24)	(n=6)	(n=6)	(n=8)	(n=11)	(n=4)	(n=5)
Amoxicillin	4 (16.6%)	23 (69.6%)	16 (64.0%)	19 (79.1%)	0(0.0%)	1(16.6%)	2 (25.0%)	6(54.5%)	1 (25.0%)	3 (60.0%)
Ceftazidime	0(0.0%)	1(3.0%)	0(0.0%)	2 (8.3%)	0(0.0%)	0(0.0%)	0(0.0%)	0(0.0%)	0(0.0%)	1 (25.0%)
Gentamicin	3(12.5%)	8 (24.2%)	0(0.0%)	1 (4.1%)	0(0.0%)	0(0.0%)	0(0.0%)	0(0.0%)	0(0.0%)	2 (40.0%)
Tetracycline	10(41.6%)	20 (60.6%)	3 (12.0%)	10 (41.6%)	0(0.0%)	0(0.0%)	0(0.0%)	2 (18.1%)	1 (25.0%)	3 (60.0%)
Nitrofurantoin	0(0.0%)	3 (9.0%)	4 (16.0%)	2 (8.3%)	0(0.0%)	0(0.0%)	0(0.0%)	0(0.0%)	1 (25.0%)	2 (40.0%)
Cotrimoxazole	4 (16.6%)	14 (42.4%)	1 (4.0%)	7 (29.1%)	0(0.0%)	0(0.0%)	0(0.0%)	1 (9.0%)	1 (25.0%)	4 (80.0%)
Nalidixic acid	2 (8.3%)	18 (54.5%)	0(0.0%)	8 (33.3%)	0(0.0%)	0(0.0%)	0(0.0%)	1 (9.0%)	3(75.0%)	3 (60.0%)

**Table 3:** Prevalence of antimicrobial resistance in isolates from raw buffalo and chicken meat samples.

Antimicrobials	Buffalo (%)	Chicken (%)	Total	*P* value
Amoxicillin	26(32.1%)	55(67.9%)	81	*0.01*
Ceftazidime*∗*	0(0.00%)	4(100%)	4	0.47
Gentamicin	3(21.4%)	11(78.6%)	14	0.05
Tetracycline	17(30.4%)	39(69.6%)	56	*0.01*
Nitrofurantoin*∗*	5(41.7%)	7(58.3%)	12	0.80
Cotrimoxazole	9(25.7%)	26(74.3%)	35	*0.01*
Nalidixic acid*∗*	5(16.7%)	30(83.3%)	35	*0.01*
Azithromycin*∗∗*	0(0.00%)	2(100%)	2	0.24
Ciprofloxacin*∗∗*	0(0.00%)	0(0.00%)	0	0.43

*Resistant to less than 3 drugs*	33(51.5%)	31(48.4%)	64	0.09
*Resistant to 3 to 4 drugs*	7(23.3%)	23(76.7%)	30	*0.01*
*Resistant to more than 4 drugs*	0(0.00%)	9(100%)	9	*0.01*

*Note*. Antimicrobials without *∗* are tested in both gram positive and gram negative isolates; antimicrobials with *∗* are tested for gram negative isolates only; antimicrobials with *∗∗* are tested for gram positive isolates only.

**Table 4:** Multidrug-resistant bacterial isolates from buffalo and chicken meat.

Bacteria	Isolates from buffalo	MDR in buffalo	Isolates from chicken	MDR in chicken	Total MDR isolates
*Escherichia coli*	24(42.1%)	7(29.1%)	33(57.8%)	23(69.6%)	30 (52.6%)
*Klebsiella *spp.	25(51.0%)	4(16.0%)	24(48.9%)	11(45.8%)	15(30.6%)
*Salmonella *spp.	6(50.0%)	0(0.0%)	6(50.0%)	0(0.0%)	0(0.0%)
*Citrobacter *spp.	8(42.1%)	0(0.0%)	11(57.9%)	2(18.1%)	2(10.5%)
*Proteus *spp.	4(44.4%)	2(50.0%)	5(55.5%)	5(100.0%)	7(77.7%)
*Staphylococcus aureus*	6(40.0%)	3(50.0%)	9(60.0%)	3(33.3%)	6(40.0%)

*Total *	*73(45.3*%)	*16(21.9*%)	*88(54.6*%)	*44(50.0*%)	*60(32.7*%)

## Data Availability

The data used to support the findings of this study are available from the corresponding author upon request. The authors agree that anyone interested to access the raw data from the research can be provided upon request to the authors. The data will be provided to anyone based on the two conditions: upon use of the data the authors should be acknowledged and also the paper needs to be cited. An agreement in the aforementioned situation will be done if agreed upon.

## Abbreviations

MDR: Multi Drug Resistant
*Spp.*: Species
CLSI: Clinical and Laboratory Standards Institute
*μ*g: Microgram
MRSA: Methicillin-Resistant* Staphylococcus aureus*.
